# CircRNAs as Potential Blood Biomarkers and Key Elements in Regulatory Networks in Gastric Cancer

**DOI:** 10.3390/ijms23020650

**Published:** 2022-01-07

**Authors:** Laís Reis-das-Mercês, Tatiana Vinasco-Sandoval, Rafael Pompeu, Aline Cruz Ramos, Ana K. M. Anaissi, Samia Demachki, Paulo Pimentel de Assumpção, Amanda F. Vidal, Ândrea Ribeiro-dos-Santos, Leandro Magalhães

**Affiliations:** 1 Laboratory of Human and Medical Genetics, Institute of Biological Sciences, Federal University of Pará, Belem 66075-110, PA, Brazil; lais.reis@icb.ufpa.br (L.R.-d.-M.); gloria.vinasco-sandoval@cea.fr (T.V.-S.); rafaelpompeu988@gmail.com (R.P.); amandaferreiravidal@gmail.com (A.F.V.); 2 Center of Oncology Research, Federal University of Pará, Belem 66073-000, PA, Brazil; nurse.alinecruz@gmail.com (A.C.R.); anakaryssa@yahoo.com.br (A.K.M.A.); demachki@gmail.com (S.D.); assumpcaopp@gmail.com (P.P.d.A.); 3 Postgraduate Program of Genetics and Molecular Biology, Institute of Biological Sciences, Federal University of Pará, Belem 66075-110, PA, Brazil

**Keywords:** gastric cancer, circRNA, microRNA, RBP, epigenetics, biomarker

## Abstract

Gastric cancer (GC) is the fifth most common type of cancer and the third leading cause of cancer death in the world. It is a disease that encompasses a variety of molecular alterations, including in non-coding RNAs such as circular RNAs (circRNAs). In the present study, we investigated hsa_circ_0000211, hsa_circ_0000284, hsa_circ_0000524, hsa_circ_0001136 and hsa_circ_0004771 expression profiles using RT-qPCR in 71 gastric tissue samples from GC patients (tumor and tumor-adjacent samples) and volunteers without cancer. In order to investigate the suitability of circRNAs as minimally invasive biomarkers, we also evaluated their expression profile through RT-qPCR in peripheral blood samples from patients with and without GC (*n* = 41). We also investigated the predicted interactions between circRNA-miRNA-mRNA and circRNA-RBP using the KEGG and Reactome databases. Overall, our results showed that hsa_circ_0000211, hsa_circ_0000284 and hsa_circ_0004771 presented equivalent expression profiles when analyzed by different methods (RNA-Seq and RT-qPCR) and different types of samples (tissue and blood). Further, functional enrichment results identified important signaling pathways related to GC. Thus, our data support the consideration of circRNAs as new, minimally invasive biomarkers capable of aiding in the diagnosis of GC and with great potential to be applied in clinical practice.

## 1. Introduction

Gastric cancer (GC) is the fifth most common type of cancer in the world, but diagnosis is usually made in late and advanced stages. This is the main reason for poor prognosis among gastric cancer patients, leading it to be the third most common cause of cancer deaths, at one in every thirteen cancer deaths in the world [1,2]. As it is a biologically heterogeneous disease, GC patients harbor several genetic and epigenetic alterations [3,4], among which we highlight the differential expression of regulatory non-coding RNAs (ncRNAs). Regulatory ncRNAs have been shown to be promising molecular markers for several diseases, including GC [5,6]. Thus, while providing a better understanding of the regulatory mechanisms carried out by these molecules, the investigation of such epigenetic elements may also lead to the discovery of new biomarkers that are sufficiently sensitive and specific, consequently improving aspects related to late diagnosis and response to treatment [7].

Circular RNAs (circRNAs) are a new class of ncRNAs that have recently gained interest. They originate through a backsplicing-like mechanism, which means that the 5′ and 3′ ends of the RNA molecule are covalently joined, resulting in a circular-shaped molecule with no free ends. This characteristic gives the molecule high stability and resistance to degradation by exonuclease RNase R [8,9]. The function of most circRNAs has not yet been fully understood [10], but studies suggest they may act as microRNA (miRNA) sponges or function through the interaction with RNA-binding proteins (RBP) [11]. Furthermore, the expression of these molecules appears to be tissue-specific or associated with developmental stages [12,13], and has been observed to be stable in body fluids such as plasma and gastric juice [14,15,16].

In recent years, scientific literature has described the global expression profile of circRNAs by microarray and next-generation sequencing (NGS), revealing hundreds of these elements with deregulated expression in GC [16,17,18]. It should be mentioned that most of these studies used adjacent to tumor tissues as non-cancer controls. However, evidence indicates that adjacent to tumor samples present molecular alterations similar to the cancer, which renders these samples as inappropriate controls for investigating differential expression profiles [18,19,20,21].

Receiver operating characteristic (ROC) curve analyses have shown that circRNAs (hsa_circ_0000190, hsa_circ_0001895, hsa_circ_0003159, and hsa_circ_0067582) have improved sensitivity, specificity and area under curve (AUC) results when compared to commonly used markers such as CEA and CA 19-9, highlighting their potential use as biomarkers [15,22,23,24]. In parallel, Chen et al. (2017) showed that hsa_circ_0000190 expression profile in the plasma was comparable to that of tissue samples from patients with gastric cancer, revealing its potential as a minimally invasive biomarker to aid in diagnosis [15]. For these characteristics, circRNAs have been considered to be promising biomarkers for this disease [14,15,22,25].

In a previous study by our group, Vidal et al. (2017) employed NGS to analyze the global expression profile of circular RNAs in three types of gastric tissue samples: GC, tumor-adjacent tissue (ADJ) and non-cancer tissue (NC). The authors found that 27 circRNAs were commonly expressed in the three types of samples. Among these, five circRNAs were upregulated in GC and ADJ when compared to NC [18]. In the present study, we validated the results observed by Vidal et al. (2017) for circRNAs hsa_circ_0000211, hsa_circ_0000284, hsa_circ_0000524, hsa_circ_0001136 and hsa_circ_0004771 through RT-qPCR using gastric tissue samples from a different group of patients. We also analyzed the expression profile of the same circRNAs in peripheral blood samples in order to investigate similarities in the expression pattern of these elements to those observed in the previous study. Our results indicate that hsa_circ_0000211, hsa_circ_0000284 and hsa_circ_0004771 can be considered to be the circRNAs with the greatest potential for becoming minimally invasive biomarkers for this disease and, therefore, with great promise to be applied in clinical practice. Such circRNAs are also important because they were already described as miRNA sponges [26,27,28,29] and as we explored their possible roles in GC, we found that they are involved in several pathways relevant to cancer development, such as apoptosis pathway and p53 signaling pathway.

## 2. Results

### 2.1. Deregulated circRNAs in GC Tissue

**Figure 1:**
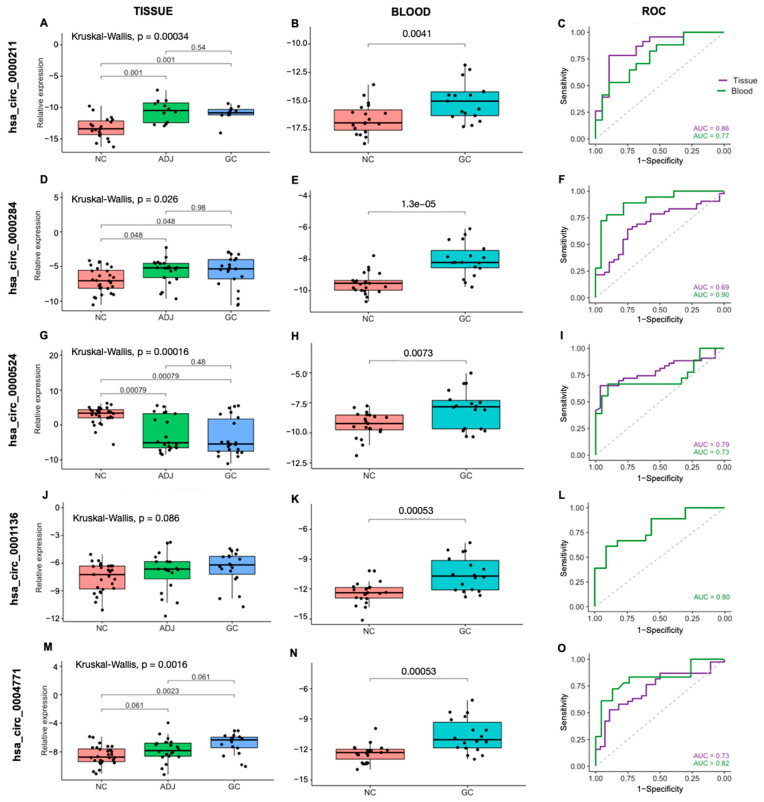
Expression profile and ROC curves for the five circRNAs in gastric tissues and blood samples. (**A**–**C**) hsa_circ_0000211, (**D**–**F**) hsa_circ_0000284, (**G**–**I**) hsa_circ_0000524, (**J**–**L**) hsa_circ_0001136 and (**M**–**O**) hsa_circ_0004771. NC: Non-cancer, GC: gastric cancer, ADJ: tumor-adjacent.

We analyzed five circRNAs in 71 gastric tissue samples (GC *n* = 22, ADJ *n* = 21, NC *n* = 28) by RT-qPCR. We found that hsa_circ_0000211, hsa_circ_0000284 and hsa_circ_0004771 were significantly upregulated in GC when compared to NC. Furthermore, hsa_circ_0000211 and hsa_circ_0000284 expression was significantly upregulated in ADJ compared with NC. Although not statistically significant, hsa_circ_0001136 showed an expression profile similar to hsa_circ_0000211, hsa_circ_0000284 and hsa_circ_0004771. On the other hand, the hsa_circ_0000524 expression was significantly downregulated in both GC and ADJ when compared to NC (*p* < 0.05; fold change < 2; Figure 1; Supplementary Table S1).

GC tissue samples were obtained more frequently from male patients and from patients under the age of 60. Regarding the Lauren’s classification, most samples in our dataset belonged to patients with the intestinal subtype followed by the diffuse subtype. We performed expression analyses considering the clinical data of patients and found that circRNAs hsa_circ_0000524 and hsa_circ_0001136 were upregulated in male and female patients, respectively. Notably, hsa_circ_0000284 and hsa_circ_0001136 were upregulated in the diffuse subtype. In addition, circRNAs hsa_circ_0000211 and hsa_circ_0000284 were upregulated in patients diagnosed at earlier stages of the disease (TNM stages I and II). We did not observe significant differences in expression profiles of circRNAs when comparing the age of patients and tumor sites (Supplementary Table S2 and Figure S1).

### 2.2. CircRNAs Are Differentially Expressed in Blood and Are Potentially Less Invasive Biomarkers for Gastric Cancer

In order to evaluate the expression profile of the five circRNAs using a minimally invasive approach, we analyzed 41 peripheral blood samples (*GC n* = 18, NC *n* = 23). The expression of all five circRNAs (hsa_circ_0000211, hsa_circ_0000284, hsa_circ_0000524, hsa_circ_0001136 and hsa_circ_0004771) was significantly upregulated in GC samples (*p* < 0.05; fold change > 2; Figure 1; Supplementary Table S1). That is, the qPCR expression profile of circRNAs hsa_circ_0000211, hsa_circ_0000284 and hsa_circ_0004771 was similar in both sample types: tissue and blood, as well as corroborating previous RNA-Seq findings; while the qPCR expression profile of circRNAs hsa_circ_0000524 and hsa_circ_0001136 found in the analysis of blood samples only corroborated previous RNA-Seq findings.

ROC curve analyses were performed to estimate the potential diagnostic value in distinguishing GC from NC individuals. We analyzed hsa_circ_0000211, hsa_circ_0000284, hsa_circ_0000524 and hsa_circ_0004771 in gastric tissue samples (GC+ADJ vs. NC), and all five circRNAs in blood samples (GC vs. NC). In both tissue and blood, the area under the ROC curve for all circRNAs was higher than 0.70, showing that these circRNAs may be considered as potential diagnostic markers, although effectiveness estimates require a larger cohort for this to be further confirmed (Figure 1; Supplementary Table S1).

In this group, cancer samples were more frequently from male patients and patients aged over 60. Expression profile analysis of circRNAs considering sex, age, surgery, *H. pylori*, Lauren’s classification and TNM staging did not reveal statistically significant differences. However, hsa_circ_0000211 and hsa_circ_0000284 were upregulated in tumors located in the antrum-pylorus region when compared to those located in the antrum body (Supplementary Table S2 and Figure S2).

### 2.3. Functional Enrichment Analyses

#### 2.3.1. CircRNA-miRNA-mRNA

A total of 75 miRNAs were found in Circular RNA Interactome with at least one predicted binding site for at least one of the five circRNAs, and according to miRTargetLink Human, 69 miRNAs have a total of 2382 genes as targets with weak and strong evidence. Of these, 24 miRNAs have 72 target genes with strong evidence (Supplementary Table S3).

**Figure 2:**
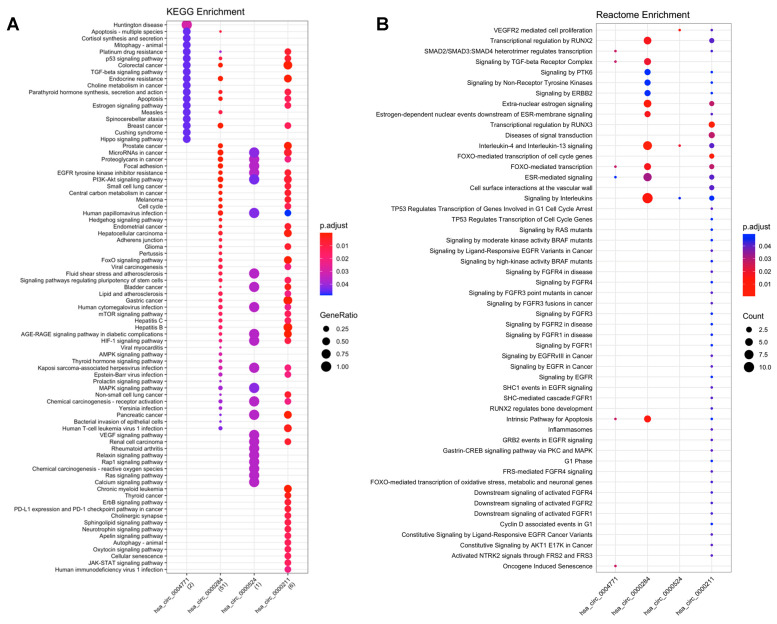
Functional enrichment analysis of circRNA-miRNA-mRNA interactions. (**A**) KEGG enrichment analysis; (**B**) Reactome enrichment analysis.

A total of 80 KEGG pathways were enriched in the analysis of miRNA-mRNA. Of these, 45 pathways were classified as human diseases, namely: 22 were related to cancer (e.g., microRNA in cancer; gastric cancer); 16 were classified as signal transduction (e.g., TGF-beta, VEGF, Ras and ErbB signaling pathways); 10 were classified as cellular processes (e.g., p53 signaling pathway; apoptosis; cell cycle and focal adhesion); and the other 9 pathways were classified as organismal class systems (nervous and endocrine systems) (Figure 2).

The Reactome enrichment generated a total of 141 biological pathways. After filtering for cancer-related pathways, we obtained 52 pathways, of which 13 are involved with diseases, including cancer (e.g., signaling by EGFRvIII in cancer; signaling by high-kinase activity BRAF mutants; signaling by RAS mutants); 21 were classified as signal transduction (e.g., signaling by PTK6; downstream signaling of activated FGFR4; signaling by EGFR; signaling by ERBB2); 11 are involved in gene expression (e.g., gastrin-CREB signaling pathway via PKC and MAPK; TP53 regulates transcription of cell cycle genes); and the remaining 7 pathways are involved with cell cycle, apoptosis, immune system, homeostasis and cellular response to external stimuli (Figure 2).

#### 2.3.2. CircRNAs-RBPs

**Figure 3:**
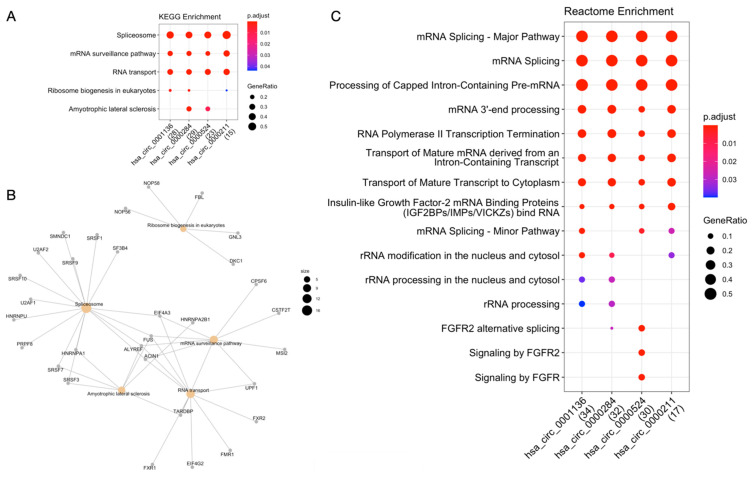
Functional enrichment analysis of circRNA-RBP interactions. (**A**) KEGG enrichment analysis. (**B**) Network of RBPs and enriched KEGG pathways. (**C**) Reactome enrichment analysis.

According to circRNA-RBP interaction data extracted from ENCORI, circRNAs hsa_circ_0000211, hsa_circ_0000284, hsa_circ_0000524 and hsa_circ_0001136 have a range between 1 and 47 binding sites for 65 different RBPs (Supplementary Table S4). KEGG enrichment analysis by RBP revealed that these genes are mostly involved in pathways of the genetic information processing class, such as transcription (e.g., spliceosome) and translation (e.g., ribosome biogenesis in eukaryotes; mRNA surveillance pathways; RNA transport) (Figure 3A). Among the 65 RBPs, we draw attention to four central RBPs identified as common in the “spliceosome”, “mRNA surveillance pathway” and “RNA transport” pathways: FUS, ALYREF, ACIN1 and EIF4A3 (Figure 3B).

Analysis of 65 RBPs with Reactome revealed 11 pathways involved in RNA metabolism (e.g., mRNA splicing; processing of capped intron-containing pre-mRNA; insulin-like growth factor-2 mRNA binding proteins—IGF2BPs/IMPs/VICKZs), 3 classified as signal transduction (e.g., signaling by FGFR) and 1 in gene expression (transcription) (RNA polymerase II transcription termination) (Figure 3C).

## 3. Discussion

Recently, a study by our group based on RNA-Seq revealed five circular RNAs (hsa_circ_0000211, hsa_circ_0000284, hsa_circ_0000524, hsa_circ_0001136 and hsa_circ_0004771) as differentially expressed in gastric cancer [18]. Subsequently, our aim in this study was to investigate and validate the expression of these circular RNAs by RT-qPCR using a larger sample number and from different types of tissues (frozen biopsies of gastric tissue and peripheral blood).

RT-qPCR analysis of tissue samples revealed that the expression profiles of circRNAs hsa_circ_0000211, hsa_circ_0000284 and hsa_circ_0004771 were upregulated in GC, in agreement with what was observed with RNA-Seq [18]. However, hsa_circ_0001136, despite following the trend of upregulation, was not significantly different in our analysis. Further, hsa_circ_0000524 did not corroborate previous data [18].

We emphasize that the deregulation of epigenetic factors is an important feature of carcinogenesis; thus, the discovery of circRNAs with aberrant expression in gastric cancer becomes even more valuable when it is likely to be observed in blood, rather than relying solely on tissue biopsy examinations, which are invasive procedures. Therefore, our efforts to investigate the expression of these five circRNAs in peripheral blood samples from patients with gastric cancer constitute an attempt to discover new minimally invasive biomarkers.

Differential expression analysis in blood samples revealed that all investigated circRNAs (hsa_circ_0000211, hsa_circ_0000284, hsa_circ_0000524, hsa_circ_0001136 and hsa_circ_0004771) were significantly upregulated in patients with gastric cancer when compared to those without cancer, validating results obtained in previous RNA-Seq analysis and using a less invasive approach. In addition, ROC curve analyses indicated that all circRNAs have good sensitivity, specificity and accuracy in distinguishing patients with gastric cancer from patients without the disease, reflecting their suitability as less invasive gastric cancer biomarker candidates.

Additionally, we correlated the expression profiles of circRNAs from tissue and blood samples from patients with gastric cancer to their respective clinicopathological data. In tissue samples, we identified that some circular RNAs were differentially expressed in the following clinical characteristics: sex, Lauren’s classification and TNM staging. Whereas in blood samples, we identified circRNAs that were differentially expressed according to tumor site. Despite the small sample number, it is noteworthy that the distribution of our samples is consistent with the epidemiological characteristics of GC, which is diagnosed in greater proportions in males [1], over 60 years old [30], with intestinal subtype (according to the Lauren’s classification) [30] and in more advanced stages of the disease [31].

There are previous reports of the circRNAs hsa_circ_0000211, hsa_circ_0000284, hsa_circ_0000524, hsa_circ_0001136 and hsa_circ_0004771 expression in various diseases; however, this is the first study to analyze and validate the circRNAs hsa_circ_0000211, hsa_circ_0000524 and hsa_circ_0001136 in gastric cancer using frozen gastric tissue and peripheral blood samples.

Hsa_circ_0000211 (circSFMBT2) is located in chr10 (7318853–7327916) and is generated by exons 5, 6 and 7 of the *SFMBT2* gene. This is the first study to report this circRNA as upregulated in GC; beyond this, there is only one report of this circRNA in the literature, in which it is shown to be upregulated in lung adenocarcinoma, acting as a sponge to miR-622 and increasing the expression of its target, *HIF-1A*, promoting cell migration and invasion. Thus, the authors suggest its use as a potential new therapeutic target for lung adenocarcinoma since aberrant cell migration could be modulated through hsa_circ_0000211 silencing in vitro [26].

Hsa_circ_0000284, or circHIPK3, is located in chr11 (33307958–33309057) and is generated by the circularization of exon 2 of the *HIPK3* gene. This exon is flanked by long introns, which contain complementary ALU repeats that help promote its circularization. Although this circRNA contains an initiation codon and is predominantly found in the cytoplasm, there is still no evidence that circHIPK3 is naturally translated [32]. The way it is reported in our study, the upregulation of circHIPK3 has been found in several types of cancer, such as: hepatocellular carcinoma (HCC) [17,33], ovarian cancer [34], colorectal cancer [35] and gastric cancer [18,27,36,37,38,39].

In GC, circHIPK3 was described as a sponge for different miRNAs. Cheng et al. (2018) identified that circHIPK3 upregulation promoted the proliferation of GC cells acting as a sponge for miRNAs miR-124 and miR-29b, and was associated with aggressive clinical factors, highly expressed in advanced tumor stages (T3 and T4) of the disease (according to the TNM staging system) and in infiltrative cell growth pattern (according to Ming’s classification). Moreover, common targets of miR-124 and miR-29b (*COL1A1*, *COL4A1* and *CDK6*) were upregulated in GC and were associated with a poor prognosis [27]. Similarly, Wei et al. (2020) identified that circHIPK3 aberrant expression was also associated with TNM stage, and that the proliferation and migration of GC cells promoted by circHIPK3 elevated expression were partially due to the regulation of the miR-107/*BNDF* axis (gene associated with the initiation and development of various types of cancers) [36].

In a study carried out in a long-term hypoxic microenvironment, circHIPK3 was upregulated under *HIF-2α* modulation and promoted the migration and invasion of GC cells through the regulation of the miR-653-5p/miR-338-3p-*NRP1* axis and its downstream pathway ERK-AKT. Furthermore, upregulation of *NRP1* was associated with a worse prognosis in GC patients. Thus, the authors suggested circHIPK3 as a possible long-term hypoxia biomarker and a potential prognostic biomarker for GC patients [37]. Liu and Xu (2019) identified that circHIPK3 elevated expression promoted proliferation and migration of GC cells through the regulation of the Wnt/β-catenin pathway (which is associated with tumor differentiation, invasion and metastasis in GC), and linked circHIPK3 upregulation to a poor GC prognosis [38].

Another study identified that circHIPK3 upregulation promoted not only proliferation and migration, but also invasion and glutaminolysis of GC cells through the regulation of the miR-876-5p/*PIK3R1* axis. By demonstrating that circHIPK3 silencing prevented proliferation, migration and invasion in vitro, and inhibited tumor growth in vivo, the authors suggested circHIPK3 as a potential future therapeutic strategy [39].

Hsa_circ_0000524 (circRBM23) is located in chr14 (23378691–23380612) and is generated by exons 4 and 5 of the *RBM23* gene. We found its expression was significantly upregulated in blood GC samples, but not in tissue. The only reports of circRBM23 in the literature are of its upregulation in both a kidney disease called membranous nephropathy [40] and in HCC [41]. In HCC, circRBM23 was able to modulate cell viability and migration in vitro, and tumorigenesis in vivo, through the miR-138/vimentin-*CCND3* axis [41].

Hsa_circ_0001136 (circASXL1) is located in chr20 (30954186–30956926) and is generated by exons 2 and 3 of the *ASXL1* gene. We found its expression was significantly upregulated in blood GC samples, but not in tissue. It has been described in essential thrombocythemia, a benign myeloproliferative disease, but with some potential to progress to acute myeloid leukemia [42]; in bladder cancer, its upregulation has been associated with more aggressive tumors (higher tumor grades, more advanced tumor stages, lymph node invasion and metastasis) and with a worse prognosis [43].

Hsa_circ_0004771, or circNRIP1, is located in chr21 (16386664–16415895) and is generated by exons 2 and 3 of the *NRIP1* gene. Its biogenesis is enhanced by the binding of RBP Quaking (QKI) to the introns that flank the exons to be circularized, and is mainly found in the cytoplasm [28]. Its expression has been investigated a few times in gastric cancer, and according to microarray results, was one of 10 circRNAs with the lowest expression patterns in the plasma of gastric cancer patients [44]. In contrast, our study as well as others has identified the upregulation of circNRIP1 in gastric cancer [28,29,45,46].

Zhang et al. (2019) identified that under RBP QKI modulation, circNRIP1 was upregulated in gastric tissue samples and exosomes (isolated from plasma) from patients with gastric cancer. The authors demonstrated that circNRIP1 acted as a miRNA sponge and that its upregulation promoted GC cell proliferation and metastasis in vitro and promoted tumor growth and metastasis in vivo, mediated by the regulation of the miR-149-5p/*AKT1* axis and consequently by the AKT/mTOR signaling pathways (responsible for metabolism alterations in gastric cancer) and EMT pathway (a promoter of tumor metastasis). Furthermore, high circNRIP1 expression has been associated with tumor size, lymph node invasion and poor prognosis [28].

Two other studies indicated that circNRIP1 acts as a miRNA sponge: in the miR-182/*ROCK1* axis by modulating migration, invasion, cell cycle progression and apoptosis of gastric cancer cells [29]; and in the miR-186-5p/*MYH9* axis by modulating proliferation, migration, glycolysis and apoptosis in vitro, and tumor volume and weight in vivo [45].

Xu et al. (2020) identified circNRIP1 upregulation in gastric cancer using tissue and plasma samples. In addition to differentiating gastric cancer patients from healthy controls, plasma circNRIP1 was able to differentiate gastric cancer patients from patients with superficial gastritis, as well as presenting dynamics of expression, decreasing from preoperative to postoperative, and increasing again when there were relapses, thus serving as a progression monitor. The upregulation of circNRIP1 was associated with poor-moderate or minimally differentiated histological differentiation, lymph node invasion, more advanced tumor stages (T3 and T4) and TNM stage (III–IV). The authors demonstrated that plasma circNRIP1 is a potential diagnostic biomarker of gastric cancer, overcoming the discriminatory capacity of CEA and CA19-9 markers, even though the detection of circNRIP1 combined with CEA and CA19-9 has surpassed the sensitivity and specificity of any biomarker detected separately [46].

To complement the results, we performed in silico analysis to shed light on the predicted biological role of these altered circRNAs through their interaction with RBPs and miRNAs (and their respective target genes), where we found important signaling pathways shared between circRNAs.

In the functional enrichment analysis of miRNA target genes, circRNAs shared signaling pathways relevant to cancer development, such as the gastric cancer pathway (and other types of cancers), microRNAs in cancer, cell cycle pathway, apoptosis pathway, p53 signaling pathway, transcriptional misregulation in cancer and others. They also shared pathways related to the studies mentioned above, such as the HIF-1 signaling pathway (involved in the regulation of oxygen homeostasis), TGF-beta, Hippo and MAPK signaling pathways (which are interconnected with the Wnt canonical pathway, involved in diverse cellular functions including proliferation, apoptosis, differentiation and migration), PI3K-Akt signaling pathway and mTOR signaling pathway (two related pathways involved in a number of key cellular processes such as cell cycle, metabolism, protein synthesis and apoptosis) and the choline metabolism in cancer and central carbon metabolism in cancer pathways (involved in the alteration of metabolism in cancer, which includes glycolysis and glutaminolysis).

Our functional analysis using data from RBP that interact with circRNAs revealed the potential involvement of circRNAs in genetic information processing pathways and RNA metabolism, which are processes related to circRNA biogenesis itself [11]. Of the pathways enriched by KEGG, we highlight the RBPs FUS, ALYREF, ACIN1 and EIF4A3. RBP FUS makes up the heterogeneous nuclear ribonucleoprotein complex (hnRNP), which is involved in pre-mRNA splicing and export of fully processed mRNA, and belongs to the FET family, which is reported to act in cellular processes such as the regulation of gene expression and processing of mRNA/microRNAs [47]. RBP ALYREF composes the TREX complex, which couples transcription to mRNA transport, and is involved in the nuclear export of spliced mRNA as an adapter [48]. RBPs ACIN1 and EIF4A3 are component proteins (auxiliary and main, respectively) of the splicing-dependent multiprotein exon junction complex (EJC), a complex responsible for marking the exon-exon junction in mRNA as a result of the splicing process; the main components remain attached to the spliced mRNAs throughout the mRNA metabolism process, influencing further processes such as nuclear export and subcellular localization [49,50].

Another example is the four RBPs (GTF2F1, HNRNPA1, PTBP1 and RBFOX2) that enriched FGFR/FGFR2 signaling pathways in Reactome. RBP GTF2F1 binds to RNA polymerase II and participates in the promotion of transcription along with GTF2B [51], while RBPs HNRNPA1, PTBP1 and RBFOX2 play roles in regulating alternative splicing [52,53,54].

Furthermore, the insulin-like growth factor-2 mRNA binding proteins (IGF2BPs/IMPs/VICKZs) binding RNA pathway was enriched for all analyzed circRNAs (hsa_circ_0000211, hsa_circ_0000284, hsa_circ_0000524 and hsa_circ_0001136) in Reactome. The proteins responsible for enriching this pathway (IGF2BP1, IGF2BP2 and IGF2BP3) are part of the family of oncofetal RNA-binding proteins that, by binding to their target mRNAs, are able to regulate their translation, stability and subcellular localization [55]. As each circRNA has multiple binding sites for each of the RBP IGF2BPs (Supplementary Table S4), it is possible that they are regulating these proteins (acting like sponges, for example), or being regulated by them (increasing stability or directing circRNA subcellular localization). In conjunction with the differential expression of circRNAs, these analyses bring new insights into the molecular mechanisms in which circRNAs may be involved.

The investigated circRNAs showed great potential to become biomarkers of GC, and further studies are needed to evaluate if they can also be used to monitor the prognostic of gastric cancer patients after surgery or chemotherapy. Due to their involvement in several pathways important to cancer, these circRNAs can also be used to develop new personalized therapies in order to modulate those pathways directly.

## 4. Materials and Methods

### 4.1. Patients and Samples

This study was approved by the Ethics Committee of the Center of Oncology Research of the Federal University of Pará (No. 1,432,512). All participants provided informed consent and this study was carried out in accordance with the principles established by the Declaration of Helsinki and the Nuremberg Code. Tissue samples were obtained from patients with gastric adenocarcinoma undergoing surgical treatment, or from cancer-free individuals undergoing a routine endoscopy process. Blood samples were obtained from patients with GC before or after they underwent the surgical procedure and samples used as controls were obtained from cancer-free individuals. Both tissue and blood samples were collected at the João de Barros Barreto University Hospital from the Federal University of Pará (UFPA). The clinical features from GC patients are detailed in Supplementary Table S2.

A total of 71 frozen tissue samples were analyzed: 43 from gastric cancer patients (22 GC samples and 21 matched ADJ samples) and 28 from volunteers without cancer and with no history of cancer (NC). After collection, samples were stored in RNAlater (Thermo Fisher Scientific, Waltham, MA, USA) and frozen in liquid nitrogen until total RNA isolation.

We also obtained 41 peripheral blood samples from different gastric cancer patients (*n* = 18) and volunteers without cancer and with no history of cancer (*n* = 23) collected in PAXgene Blood RNA Tubes (Qiagen, Hilden, Germany) following the manufacturer’s instructions and stored vertically at −70 °C until total RNA isolation.

### 4.2. RNA Isolation

Total RNA isolation was performed using a TRIzol reagent kit (Invitrogen, Waltham, MA, USA) for tissue samples and PAXgene Blood miRNA Kit (Qiagen, Hilden, Germany) for blood samples, both following the protocol provided by the manufacturer. Sample quality and quantification were assessed with Qubit 2.0 Fluorometer (Thermo Fisher Scientific, Waltham, MA, USA) and NanoDrop ND-1000 (Thermo Fisher Scientific, Waltham, MA, USA).

### 4.3. Reverse Transcriptase Quantitative PCR (RT-qPCR)

RNA was reverse transcribed into cDNA from 8 ng (frozen tissue) and 50 ng (blood) of total RNA using random primers and SuperScript III First-Strand Synthesis kit (Thermo Fisher Scientific, Waltham, MA, USA), following the manufacturer’s instructions.

The qPCR was performed on ABI PRISM 7500 using SsoAdvanced™ Universal SYBR^®^ Green SuperMix (Bio-Rad, Hercules, CA, USA) with specific primers (listed in Supplementary Table S5). Reactions consisted of 1 µL of cDNA, 500 nM of each forward and reverse primer, 5 µL of qPCR Master Mix and 3 µL of nuclease-free water in thermal cycling conditions provided by the manufacturer. All reactions were performed in triplicates and ß-actin was used as an endogenous control. RT-qPCR data were analyzed using the comparative Ct Method [56].

### 4.4. Functional Enrichment Analyses

CircRNA-miRNA and miRNA-mRNAs interactions were predicted using the Circular RNA Interactome (https://circinteractome.nia.nih.gov, accessed on 6 October 2021) and miRTargetLink Human (https://ccb-web.cs.uni-saarland.de/mirtargetlink/, accessed on 6 October 2021) online tools, respectively. Furthermore, circRNA-RBP interaction data were extracted from ENCORI (http://starbase.sysu.edu.cn, accessed on 5 September 2021), a CLIP-seq confirmed RNA interaction platform. This method (CLIP-seq) adds more confidence to the data since analyses are not based on prediction algorithms.

Enrichment analyses of the circRNA interactions were conducted with KEGG and Reactome pathways using ClusterProfiler [57] and ReactomePA [58] packages in R software (version 4.0.2, R Foundation, Vienna, Austria). All graphs were made using R (version 4.0.2, R Foundation, Vienna, Austria) and interaction networks of the sponged RBPs and enriched pathways were constructed using the cnetplot() function implemented in R. Enriched terms with an FDR adjusted *p*-value < 0.05 were considered to be statistically significant.

### 4.5. Statistical Analyses

For gastric tissue samples, circRNA expression levels were compared between groups using Kruskal–Wallis multiple comparison with FDR adjusted *p*-values. CircRNA expression profiles in blood samples were compared between patients and controls using Student’s *t*-test.

ROC curves were performed to evaluate the biomarker potential of the studied circular RNAs, which informs the sensitivity, specificity and AUC that each molecule has in discriminating two groups. All statistical analyses as well as graphs were performed in R (version 4.0.2, R Foundation, Vienna, Austria) using RStudio (version 1.3.1073, RStudio, Boston, MA, USA) interface. *p*-values < 0.05 were considered to be statistically significant.

## 5. Conclusions

Three circRNAs, hsa_circ_0000211, hsa_circ_0000284 and hsa_circ_0004771, which showed a uniform expression pattern in the RNA-Seq results and in the validation by RT-qPCR (in frozen gastric tissue and peripheral blood samples), were shown to be important potential biomarkers for gastric cancer. Additionally, considering the expression profile of circRNAs and their high capacity to discriminate patients with and without cancer, as well as their interaction processes (circRNAs-miRNAs-mRNAs and circRNAs-RBPs), our results suggest a strong involvement of these circRNAs in the regulation of a series of cellular processes in gastric carcinogenesis, in addition to highlighting them as excellent candidates both for new therapeutic targets and for their use as new, minimally invasive biomarkers capable of aiding in diagnosis and management of GC.

## Data Availability

The data presented in this study are available on request from the corresponding authors.

## Supplementary Materials

The following are available online at https://www.mdpi.com/article/10.3390/ijms23020650/s1.
