# Supplementary material for: CircRNAs as Potential Blood Biomarkers and Key Elements in Regulatory Networks in Gastric Cancer
Source: Int J Mol Sci. 2022 Jan 7;23(2):650. doi: 10.3390/ijms23020650 (PMC8776217; doi:10.3390/ijms23020650)
Supplement: Supplementary file 1 [file ijms-23-00650-s001.zip › ijms-1500455-supplementary.pdf]

International Journal of *Molecular Sciences*

MDPI

Supplementary materials

CircRNAs as Potential Blood Biomarkers and Key Elements in Regulatory Networks in Gastric Cancer

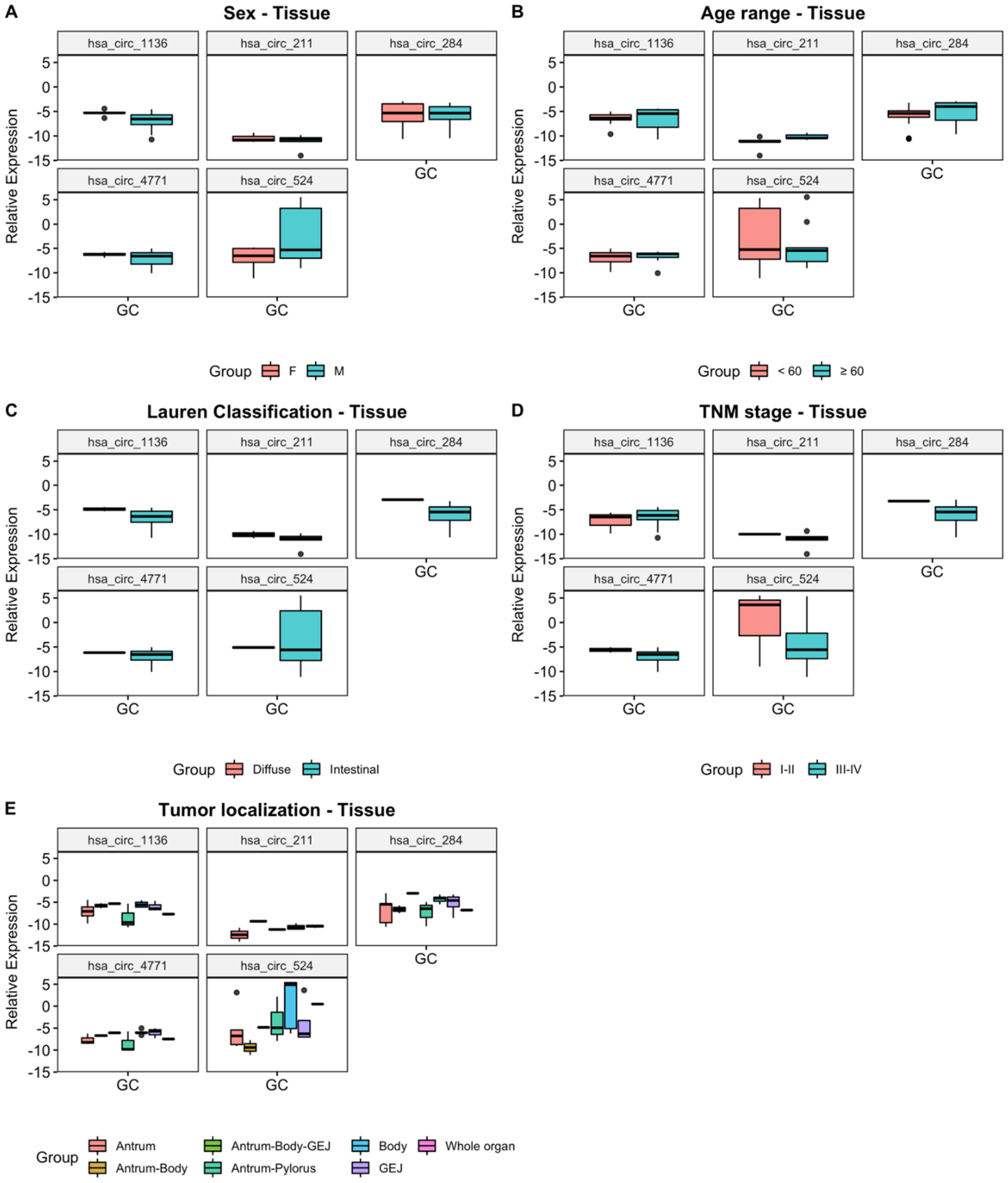

Figure S1. Correlation of circRNAs expression in gastric cancer tissue samples with clinicopathological data investigated in patients with gastric adenocarcinoma. GC: gastric cancer.

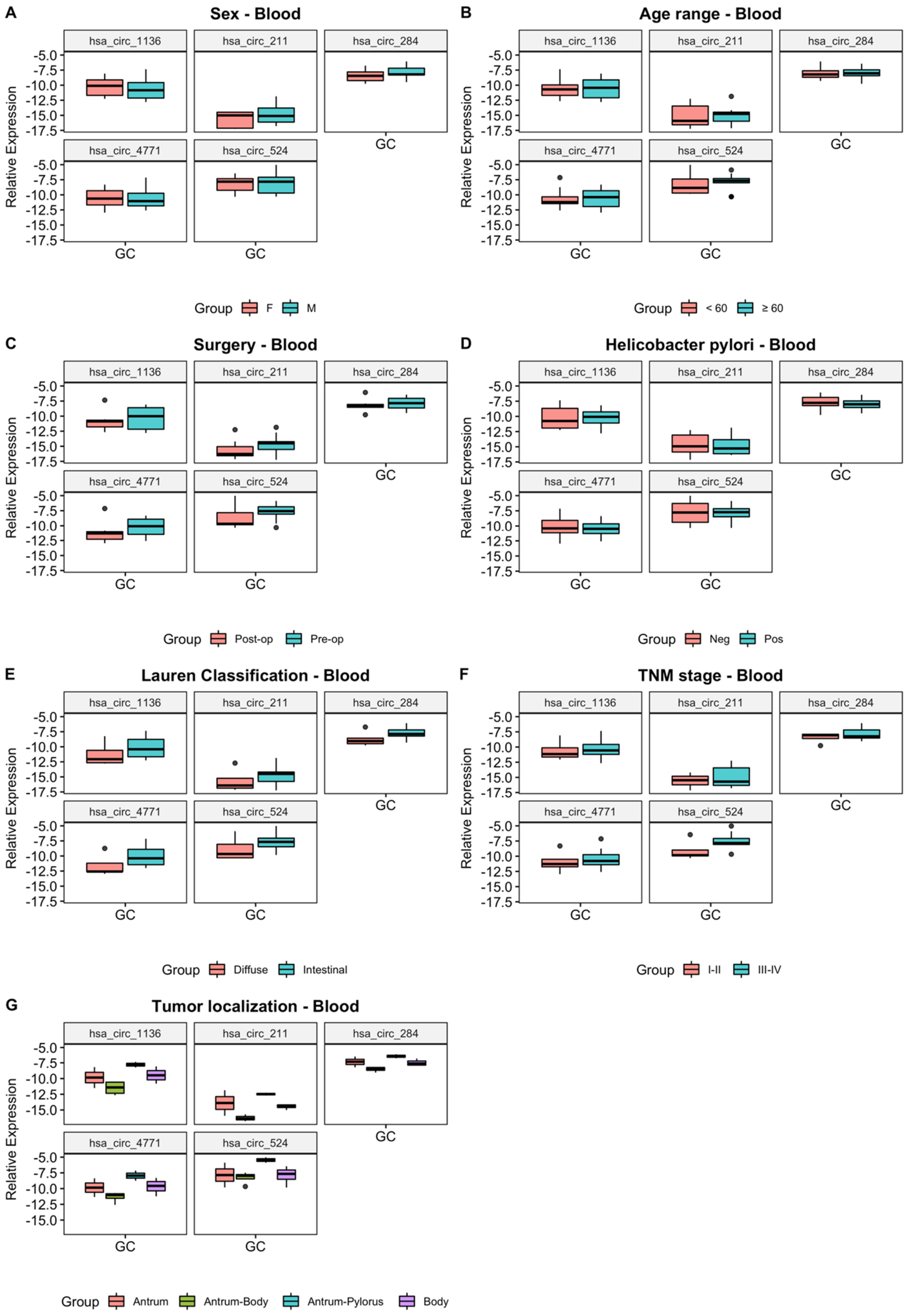

Figure S2. Correlation of circRNAs expression in gastric cancer blood samples with clinicopathological data investigated in patients with gastric adenocarcinoma. GC: gastric cancer.

Table S1. Expression and accuracy profile (AUC) analysis of circular RNAs in frozen gastric tissue and peripheral blood samples from patients with gastric adenocarcinoma and cancer-free individuals.

Circular RNAs	Sample Type	Shapiro-Wilk			*p* Values<br>Kruskal-Wallis	Wilcoxon (tissue) or T-Test (blood)			Fold Change			AUC
		NC	GC	ADJ	NC/GC/ADJ	GC vs. NC	ADJ vs. NC	GC vs. ADJ	GC vs. NC	ADJ vs. NC	GC vs. ADJ	CG+ADJ vs. NC
hsa_circ_0000211	Tissue	0.99	0.01	0.43	0.0003	0.001	0.001	ns	5.23	6.55	–1.25	0.86
	Blood	0.85	0.28	-	-	0.004	-	-	2.96	-	-	0.77
hsa_circ_0000284	Tissue	0.46	0.04	0.05	0.026	0.048	0.048	ns	2.34	2.55	–1.09	0.69
	Blood	0.36	0.67	-	-	1.3×10^{-5}	-	-	2.84	-	-	0.90
hsa_circ_0000524	Tissue	0.004	0.01	0.003	0.0002	0.0008	0.001	ns	–83.95	–40.39	–2.08	0.79
	Blood	0.44	0.21	-	-	0.0073	-	-	2.43	-	-	0.73
hsa_circ_0001136	Tissue	0.21	0.02	0.17	ns	-	-	-	2.04	1.53	1.33	-
	Blood	0.42	0.17	-	-	0.0005	-	-	3.61	-	-	0.80
hsa_circ_0004771	Tissue	0.79	0.04	0.96	0.002	0.0023	ns	ns	3.19	1.73	1.84	0.73
	Blood	0.17	0.35	-	-	0.0005	-	-	3.35	-	-	0.82

NC: Non cancer, GC: gastric cancer, ADJ: tumor-adjacent.

Table S2. Analyses of clinicopathological data investigated in patients with gastric adenocarcinoma.

Clinical Features		Tissue Samples (%)		*p*-Values *					Blood Samples (%)		*p*-Values *				
Sex	Female	6	(27.3%)	0.49^{Δ}	1.0^{Δ}	0.014^{Δ}	0.014^{Δ}	0.14^{Δ}	6	(33.3%)	0.27^{Δ}	0.31^{Δ}	0.74^{Δ}	0.7^{Δ}	0.96^{Δ}
	Male	16	(72.7%)						12	(66.7%)					
Age	<60	12	(54.5%)	0.08^{Δ}	0.54^{Δ}	0.34^{Δ}	0.9^{Δ}	0.9^{Δ}	8	(44.4%)	0.92^{Δ}	0.95^{Δ}	0.68^{Δ}	1.0^{Δ}	0.98^{Δ}
	≥60	10	(45.4%)						10	(55.6%)					
Surgery	Pre-op	-		-	-	-	-	-	11	(61.1%)	0.27^{Δ}	0.64^{Δ}	0.27^{Δ}	0.53^{Δ}	0.31^{Δ}
	Post-op	-							7	(38.9%)					
H. pylori	Negative	-							6	(33.3%)					
	Positive	-		-	-	-	-	-	4	(22.2%)	0.97^{Δ}	0.76^{Δ}	0.92^{Δ}	0.97^{Δ}	0.8^{Δ}
	NA **	22	(100%)						8	(44.4%)					
Lauren's classification	Intestinal	20	(90.9%)						12	(66.7%)					
	Diffuse	2	(9.1%)	0.4^{Δ}	$1.4\times 10^{-5\,\Delta}$	0.21^{Δ}	0.044^{Δ}	0.61^{Δ}	5	(27.8%)	0.43^{β}	0.2^{β}	0.44^{β}	0.52^{β}	0.28^{β}
	Indeterminate	-							1	(5.5%)					
Tumor location	Antrum	6	(27.3%)						2	(11.1%)					
	Antrum-Body	2	(9.1%)						4	(22.2%)					
	Antrum-Body-GEJ	1	(4.5%)						-						
	Antrum-Pylorus	3	(13.6%)	0.25^{β}	0.45^{β}	0.29^{β}	0.33^{β}	0.21^{β}	2	(11.1%)	0.035^{∂}	0.018^{∂}	0.23^{β}	0.057^{β}	0.075^{β}
	Body	5	(22.7%)						4	(22.2%)					
	GEJ	4	(18.2%)						-						
	Whole organ	1	(4.5%)						-						
	NA **	-							6	(33.3%)					
TNM stage	I-II	3	(13.6%)						4	(22.2%)					
	III-IV	19	(86.4%)	0.042^{Δ}	$6.2\times 10^{-5\,\Delta}$	0.45^{Δ}	0.56^{Δ}	0.16^{Δ}	8	(44.4%)	0.49^{Δ}	0.34^{Δ}	0.17^{Δ}	0.79^{Δ}	0.65^{Δ}
	NA **	-							6	(33.3%)					

* hsa_circ_0000211, hsa_circ_0000284, hsa_circ_0000524, hsa_circ_0001136 and hsa_circ_0004771 *p*-values respectively. ** Data disregarded in statistical analysis. NA = Not available; GEJ = Gastroesophageal junction. Δ: T-test; β: ANOVA; ∂: TUKEY HSD when comparing "Antrum-Pylorus" vs. "Antrum-Body".

Table S3. Potential circRNAs-miRNAs-mRNAs interactions.

Circular RNA	Sites	microRNAs	Target Genes with Strong Evidence
hsa_circ_0000211	1	hsa-miR-182-5p	*BCL2* *CDKN1A* *SMAD4*
hsa_circ_0000211	1	hsa-miR-188-3p	
hsa_circ_0000211	1	hsa-miR-198	
hsa_circ_0000211	1	hsa-miR-224-5p	*BCL2* *KRAS* *SMAD4*
hsa_circ_0000211	1	hsa-miR-582-3p	
hsa_circ_0000211	1	hsa-miR-587	
hsa_circ_0000211	1	hsa-miR-622	*KRAS*
hsa_circ_0000211	1	hsa-miR-626	*SLC7A5*
hsa_circ_0000211	1	hsa-miR-665	*CD274*
hsa_circ_0000211	1	hsa-miR-7	*BCL2* *SLC7A5*
hsa_circ_0000211	1	hsa-miR-885-3p	*CD274*
hsa_circ_0000211	1	hsa-miR-942	*CDKN1A*
hsa_circ_0000211	1	hsa-miR-1278	
hsa_circ_0000211	1	hsa-miR-1290	
hsa_circ_0000211	1	hsa-miR-1296	
hsa_circ_0000211	1	hsa-miR-1305	
hsa_circ_0000284	1	hsa-miR-149	*FGFR1* *SP1*
hsa_circ_0000284	1		*ACVR2B, ALCAM, BCL2, CAV1, CDC7, CUL5,*
hsa_circ_0000284	1		*DICER1, DLG5, DTL, ERCC3, ERCC4, HRH1,*
hsa_circ_0000284	1	hsa-miR-192	*HOXA10, KIF20B, LMNB2, MAD2L1, MCM10, MIS12,*
hsa_circ_0000284	1		*PIM1, PRPF38A, RACGAP1, RB1, SEPT10, SMARCB1,*
hsa_circ_0000284	1		*WNK1*
hsa_circ_0000284	1	hsa-miR-215	*ACVR2B, ALCAM, CTNNBIP1, RB1, WNK1, ZEB2*
hsa_circ_0000284	1	hsa-miR-326	*NOB1, NOTCH1, NOTCH2, PKM, SMO*
hsa_circ_0000284	1	hsa-miR-330-5p	
hsa_circ_0000284	2	hsa-miR-338-3p	*CCND1* *SMO* *UBE2Q1* *ZEB2*
hsa_circ_0000284	1	hsa-miR-346	*GSK3B* *LIF*
hsa_circ_0000284	1		*BCL2, BCL2L11, CAB39, CASP3, ELAVL4, ERBB2,*
hsa_circ_0000284	1	hsa-miR-375	*IGF1R, ITGB1, LDHB, LEPROTL1, MTDH, MTPN,*
hsa_circ_0000284	1		*PARP1, PHLPP1, RAB10, RHOA, SP1, TIMM8A, USP1,*
hsa_circ_0000284	1		*YWHAZ*
hsa_circ_0000284	1	hsa-miR-377	*PPM1A* *SOD2*
hsa_circ_0000284	1	hsa-miR-382	*PTEN*
hsa_circ_0000284	1	hsa-miR-485-3p	*MAT1A*
hsa_circ_0000284	1	hsa-miR-490-5p	*FOS*
hsa_circ_0000284	1	hsa-miR-495	*MAT1A* *PTP4A3* *SOX9*
hsa_circ_0000284	1	hsa-miR-508-3p	
hsa_circ_0000284	1	hsa-miR-513a-3p	
hsa_circ_0000284	1	hsa-miR-515-5p	
hsa_circ_0000284	1	hsa-miR-558	
hsa_circ_0000284	1	hsa-miR-561	
hsa_circ_0000284	1	hsa-miR-579	
hsa_circ_0000284	1	hsa-miR-580-3p	*TWIST1*
hsa_circ_0000284	1	hsa-miR-584	
hsa_circ_0000284	1	hsa-miR-599	
hsa_circ_0000284	1	hsa-miR-606	
hsa_circ_0000284	1	hsa-miR-607	
hsa_circ_0000284	1	hsa-miR-619	
hsa_circ_0000284	1	hsa-miR-637	

hsa_circ_0000284	1	hsa-miR-640		
hsa_circ_0000284	2	hsa-miR-653		
hsa_circ_0000284	1	hsa-miR-668		
hsa_circ_0000284	1	hsa-miR-766		
hsa_circ_0000284	1	hsa-miR-1178		
hsa_circ_0000284	1	hsa-miR-1179		
hsa_circ_0000284	2	hsa-miR-1231		
hsa_circ_0000284	1	hsa-miR-1243		
hsa_circ_0000284	1	hsa-miR-1250		
hsa_circ_0000284	1	hsa-miR-1278		
hsa_circ_0000284	1	hsa-miR-1283		
hsa_circ_0000284	1	hsa-miR-1286		
hsa_circ_0000284	1	hsa-miR-1290		
hsa_circ_0000284	1	hsa-miR-1294		
hsa_circ_0000284	2	hsa-miR-1305		
hsa_circ_0000284	1	hsa-miR-1825		
hsa_circ_0000524	1	hsa-miR-338-3p		
hsa_circ_0000524	1	hsa-miR-520g	*VEGFA*	
hsa_circ_0000524	1	hsa-miR-520h	*VEGFA*	
hsa_circ_0000524	1	hsa-miR-876-3p		
hsa_circ_0000524	1	hsa-miR-1178		
hsa_circ_0000524	1	hsa-miR-1208		
hsa_circ_0001136	1	hsa-miR-576-5p		
hsa_circ_0001136	1	hsa-miR-616		
hsa_circ_0001136	1	hsa-miR-767-3p	*MGMT*	
hsa_circ_0001136	1	hsa-miR-1179		
hsa_circ_0001136	1	hsa-miR-1244		
hsa_circ_0001136	1	hsa-miR-1248		
hsa_circ_0001136	1	hsa-miR-1304		
hsa_circ_0001136	1	hsa-miR-1827		
hsa_circ_0004771	1	hsa-miR-149	*BBC3*	*SP1*
hsa_circ_0004771	1	hsa-miR-515-5p		
hsa_circ_0004771	1	hsa-miR-532-3p		
hsa_circ_0004771	1	hsa-miR-562		
hsa_circ_0004771	1	hsa-miR-580-3p		
hsa_circ_0004771	1	hsa-miR-589		
hsa_circ_0004771	1	hsa-miR-595		
hsa_circ_0004771	1	hsa-miR-648		
hsa_circ_0004771	1	hsa-miR-653		
hsa_circ_0004771	1	hsa-miR-924		
hsa_circ_0004771	1	hsa-miR-1200		
hsa_circ_0004771	1	hsa-miR-1203		
hsa_circ_0004771	1	hsa-miR-1253		

Table S4. Potential circRNAs-RBPs interactions.

RBP	Circular RNA	Site
ACIN1	hsa_circ_0000211, hsa_circ_0001136	5, 7
ALYREF	hsa_circ_0000284, hsa_circ_0001136	2, 1
BUD13	hsa_circ_0000284, hsa_circ_0001136	3, 4
CAPRIN1	hsa_circ_0000284	1
CNBP	hsa_circ_0000211, hsa_circ_0000284, hsa_circ_0001136	2, 7, 3
CPSF6	hsa_circ_0000524, hsa_circ_0001136	2, 1
CSTF2T	hsa_circ_0000284, hsa_circ_0001136	1, 1
DDX3X	hsa_circ_0000284, hsa_circ_0000524, hsa_circ_0001136	3, 4, 2
DDX54	hsa_circ_0000211, hsa_circ_0000284, hsa_circ_0000524, hsa_circ_0001136	2, 20, 3, 6
DGCR8	hsa_circ_0000524, hsa_circ_0001136	1, 1
DICER1	hsa_circ_0000284	1
DKC1	hsa_circ_0000284, hsa_circ_0001136	1, 1
EIF4A3	hsa_circ_0000211, hsa_circ_0000284, hsa_circ_0000524, hsa_circ_0001136	3, 10, 2, 4
EIF4G2	hsa_circ_0000524, hsa_circ_0001136	1, 1
ELAVL1	hsa_circ_0000284, hsa_circ_0000524, hsa_circ_0001136	3, 2, 2
ELAVL3	hsa_circ_0000284	2
FAM120A	hsa_circ_0000284	6
FBL	hsa_circ_0000211, hsa_circ_0000284, hsa_circ_0000524, hsa_circ_0001136	5, 5, 1, 3
FMR1	hsa_circ_0000211, hsa_circ_0000284, hsa_circ_0000524, hsa_circ_0001136	7, 35, 6, 9
FUS	hsa_circ_0000211, hsa_circ_0000284, hsa_circ_0000524	1, 6, 1
FXR1	hsa_circ_0000284, hsa_circ_0000524	7, 1
FXR2	hsa_circ_0000284, hsa_circ_0001136	3, 1
GNL3	hsa_circ_0000284	2
GTF2F1	hsa_circ_0000524	1
HNRNPA1	hsa_circ_0000284, hsa_circ_0000524	2, 1
HNRNPA2B1	hsa_circ_0000284, hsa_circ_0000524, hsa_circ_0001136	2, 4, 1
HNRNPU	hsa_circ_0000524	2
IGF2BP1	hsa_circ_0000211, hsa_circ_0000284, hsa_circ_0000524, hsa_circ_0001136	3, 15, 2, 5
IGF2BP2	hsa_circ_0000211, hsa_circ_0000284, hsa_circ_0000524, hsa_circ_0001136	14, 31, 4, 14
IGF2BP3	hsa_circ_0000211, hsa_circ_0000284, hsa_circ_0000524, hsa_circ_0001136	2, 6, 1, 2
KHDRBS2	hsa_circ_0000284, hsa_circ_0000524, hsa_circ_0001136	1, 1, 1
LIN28A	hsa_circ_0000284, hsa_circ_0001136	3, 1
LIN28	hsa_circ_0000284, hsa_circ_0000524, hsa_circ_0001136	3, 2, 1
LIN28B	hsa_circ_0000284, hsa_circ_0000524, hsa_circ_0001136	11, 1, 6
MBNL2	hsa_circ_0000284	1
METTL3	hsa_circ_0001136	1

MOV10	hsa_circ_0000211, hsa_circ_0000284, hsa_circ_0000524, hsa_circ_0001136	11, 47, 1, 11
MSI2	hsa_circ_0000211, hsa_circ_0000284	2, 3
NOP56	hsa_circ_0001136	2
NOP58	hsa_circ_0000211, hsa_circ_0000284, hsa_circ_0000524, hsa_circ_0001136	6, 5, 2, 3
NPM1	hsa_circ_0000284	1
NUMA1	hsa_circ_0000524	2
PRPF8	hsa_circ_0000211, hsa_circ_0001136	1, 3
PTBP1	hsa_circ_0000211, hsa_circ_0000284, hsa_circ_0000524, hsa_circ_0001136	1, 3, 4, 2
RBFOX2	hsa_circ_0000524	4
RBM5	hsa_circ_0000524, hsa_circ_0001136	1, 1
RBM10	hsa_circ_0000211, hsa_circ_0000284, hsa_circ_0000524, hsa_circ_0001136	7, 3, 4, 4
RBM47	hsa_circ_0000284, hsa_circ_0001136	4, 1
SF3B4	hsa_circ_0001136	2
SLTM	hsa_circ_0000284	1
SMNDC1	hsa_circ_0000284, hsa_circ_0000524, hsa_circ_0001136	1, 1, 2
SND1	hsa_circ_0000284, hsa_circ_0000524	2, 2
SRSF1	hsa_circ_0000211, hsa_circ_0000284, hsa_circ_0000524, hsa_circ_0001136	4, 9, 7, 9
SRSF3	hsa_circ_0000211, hsa_circ_0000284	1, 4
SRSF7	hsa_circ_0000284, hsa_circ_0000524, hsa_circ_0001136	2, 1, 2
SRSF9	hsa_circ_0000284, hsa_circ_0001136	2, 2
SRSF10	hsa_circ_0000211, hsa_circ_0000284, hsa_circ_0000524, hsa_circ_0001136	1, 1, 1, 1
TAF15	hsa_circ_0000211, hsa_circ_0000284	1, 2
TARDBP	hsa_circ_0000284, hsa_circ_0000524, hsa_circ_0001136	1, 2, 1
U2AF1	hsa_circ_0001136	3
U2AF2	hsa_circ_0000211, hsa_circ_0000284, hsa_circ_0000524, hsa_circ_0001136	11, 4, 2, 8
UPF1	hsa_circ_0000211, hsa_circ_0000284, hsa_circ_0000524, hsa_circ_0001136	5, 8, 3, 3
YTHDC1	hsa_circ_0000284	2
YTHDF1	hsa_circ_0000284	1
YWHAG	hsa_circ_0001136	2

Table S5. Primers sequences used for qRT-PCR.

RNA Circular	Type	Forward Primer	Reverse Primer	Product Size (pb)
hsa_circ_0000211	Spanning junction divergent primer	5′ GGAAGTGTTTAAGCAATCAAAG 3′	5′ TTCCTCCAACATTTTCAATC 3′	220
hsa_circ_0000284	Divergent primer	5′ TATGTTGGTGGATCCTGTTCGGCA 3′	5′ TGGTGGGTAGACCAAGACTTGTGA 3′	146
hsa_circ_0000524	Spanning junction divergent primer	5′ GGGGAGACAAGCAAATCTGAC 3′	5′ CCTTTGTTGCTCATCCTCTTCT 3′	105
hsa_circ_0001136	Spanning junction divergent primer	5′ CCTTTTCACGCTCAAGGTATTAG 3′	5′CTGCTCATTTCCTTTAGTCCTTCT 3′	102
hsa_circ_0004771	Divergent primer	5′ CAGCCTTCTCAATTTTCTTTCTCAG 3′	5′ GCTGTGTTTCTCCCAAATGTT 3′	130
ACTB	Convergent primer	5′ GCTCGTCGTCGACAACGGCTC 3′	5′ CAAACATGATCTGGGTCATCTTCTC 3′	487
